# Embedding Passive Monitoring Into Global Health and Longitudinal Patient Care

**DOI:** 10.2196/81580

**Published:** 2025-12-30

**Authors:** Angelika Papanicolaou, Laura Gaetano, Olivia Yu, Graham Jones

**Affiliations:** 1 Neuroscience Development, Novartis AG, Basel, Switzerland; 2 Clinical Innovation, Novartis Pharmaceuticals, San Diego, CA, United States; 3 Tufts University Medical Center, 800 Washington StreetBoston, MA, 02115, United States, 1 857 2757045

**Keywords:** remote monitoring, digital health technologies, global health, patient data, health data, smart ring

## Abstract

A multitude of digital health tools have been developed to monitor, record, and predict health-related events in healthy subjects and patients. In clinical settings, although promising advances have resulted in near-term benefits, their use in longer-term studies is often limited due to the level of friction and burden imposed on the subject, often requiring active engagement by the patient with digital devices and/or its interfaces. Herein, we outline how smart ring technologies could form the anchor point for passive patient monitoring systems by offering a near-ideal compromise between device form factor and data capturing capacity. By using wireless technologies, such devices could form integral components of a hub-and-spoke health monitoring system, feeding data to cloud-based patient electronic health records and allowing push–pull actions through bidirectional communication. Such capabilities could have immediate utility in the longitudinal monitoring of patients diagnosed with slow progressing disease such as cardiovascular and neurodegenerative conditions. Moreover, if integrated through provisioned federated wireless networks, the technology could become components of global health care. To be successful, such a grand challenge would naturally require multiple technological, financial, and data privacy obstacles to be overcome. In support of this vision, we outline practical considerations for the development of such systems for specific applications and potential next steps for implementation.

## Introduction

Digital health technologies have developed rapidly over the past decade, fueled by dramatic improvements in the capability of miniaturized sensors coupled with ever increasing network and computing power [1]. This has resulted in a wide range of options, from commodity-scale consumer-worn products to elaborate clinical grade devices able to track and monitor multiple health-related signals [2]. Although a vast majority of these devices are deployed as performance trackers by healthy individuals motivated to monitor and assess specific personal targets (eg, step counting, heart rate, or sleep cycles), there is increasing use in clinical trials to enable study coordinators to monitor patient data through a combination of passive signals and patient-reported outcomes [3]. For short-term studies, this can be an extremely valuable approach, allowing the gathering of data in real-world environments and the potential for continual monitoring to augment episodic evaluations by physicians. For longer-term studies, however, patient adherence and persistence using the devices often wanes over time, rendering the data unreliable, and this is exacerbated in studies that utilize multiple devices [4]. For these reasons, there is growing interest in the use of fully passive monitoring devices, where following setup, little or no engagement is necessary [5]. For tightly controlled clinical studies, where costs are borne by sponsors, this has opened up new vistas for the development of highly sophisticated systems that provide high-precision clinical-grade data longitudinally [6]. Though attractive, for population-level studies and particularly those in developing nations, economic considerations likely preclude such options. In such instances, a compromise among device cost, simplicity, and features (ie, durability, precision, reporting frequency, and patient affinity) needs to be considered, and recent developments in smart ring technology render them well suited to address a variety of uses [7]. Representing a near-minimal form-factor for a personal wearable, contemporary smart rings now offer a wide range of health-sensing functions ranging from heart rate, actigraphy, sleep, and temperature derived from algorithms that are continually refined and updated [8-10]. Although smart rings are far from clinical grade standards, they can provide useful information; moreover, owing to form factors that blend well (thus not signaling to onlookers that the person may be wearing a medical device) and good battery life, which requires only periodic charging, they offer a viable and patient friendly approach to long-term health monitoring. Data have the potential to be accessed from the cloud, via interfaces to smart phones, or through connection to a device reader, giving considerable flexibility to the level of patient engagement and burden. Extrapolating further, one could envision the deployment of such devices as fully passive autonomous monitors, providing cloud-based periodic or real-time updates on clinical trial metrics, or in the case of long-term monitoring of slow progressing diseases (eg, cardiometabolic or neurodegenerative), forming diarized components of the patient’s electronic health record [11-13]. Herein, we outline how such systems could become embedded in personal health tracking, potential considerations for adoption, and mechanisms to incentivize deployment in various components of the health care ecosystem.

## Discussion

### Overview

**Figure 1.**
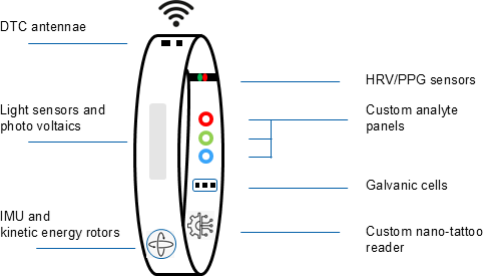
Hypothetical components of a ring-based passive monitor. DTC: direct to cloud HRV=heart rate variability; IMU: inertial measurement unit; PPG: photoplethysmography.

The increased miniaturization of health-based electronic sensors potentially allows myriad monitoring instruments to be embedded in a single device. Among recent developments, consumer smart rings now host a number of sophisticated analytical tools, reflecting a compromise between cost and functionality (Figure 1). These typically include photoplethysmography/pulse shape measurement, temperature, and motion/inertial measurement unit sensors. There are also numerous prototype sensors in development that have shown promise for custom analyte detection, which might be incorporated into ring architectures including galvanic monitors [14], nano-tattoo–based circuits [15], and custom analytic cells [16]. For powering these devices, there is the potential to extend duty cycles through the use of solar cell technology and a wide range of emerging options for wireless data transmission from such devices. Although the commercial focus lies in the development of these products for the direct-to-consumer market, an attractive proposition could be to deploy a standardized version for use at scale in global health. This would require that data and device standards be identified, and a minimum set of sensors and functionality be established for patient monitoring. This could, for example, reflect current aggregated health care needs of national priority (eg, cardiovascular monitoring) or sensors fine tuned for regional needs including epidemic, pandemic, or episodic flares of specific diseases where tracking is important.

**Figure 2.**
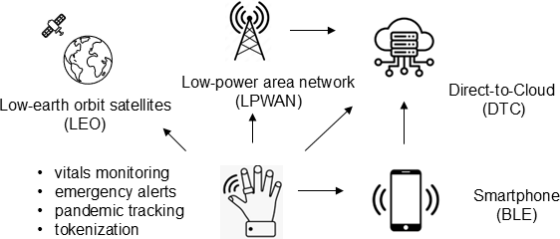
Wireless networking options through ring-based passive monitors. BLE: bluetooth low energy.

In terms of connectivity, various options exist for wireless-based communication between the device and a data hub/host. The most straightforward of these is via a Bluetooth link to a nearby smartphone, with the option to bounce data directly to the cloud (Figure 2). In regions where 4G and 5G networks are available, this could utilize direct-to-cloud connectivity through 802.11 Wi-Fi protocols on local area networks (2.4 GHz band). However, on the global scale where network infrastructure is limited, this might capitalize on low-power area networks (LoRaWAN), which have been proven successful in a number of nations, allowing data to be transmitted in defined packets at intervals [17]. Yet another option could be through connection to the new low-orbit satellite–based networks being developed such as Star Link or the Inmarsat system [18]. In theory, a multitude of options could be made available, with the possibility of a standardized smart ring capable of communicating with a data hub anywhere in the world at any time. Data collected or stored would be ideally linked to databases formatted for electronic health records to allow passive, real-time longitudinal monitoring of people affiliated with country-specific health systems, or patients as they show progress as engaged users of the health system. Examples of such monitoring could include the monitoring of vital signs, providing emergency alerts to health providers (and tied to global system for mobile communication (GSM) location–based tracking for emergency responders), and monitoring of mobility (eg, during a pandemic crisis). Clearly, any approach of this nature would require compliance with health data privacy architectures including provisions regulated by bodies such as HIPAA (Health Insurance Portability and Accountability Act), GDPR (General Data Protection Regulation), and the World Health Organization but the benefits could justify such a challenge [19].

### Incentivizing and Monetizing

The ultimate goal of developing a standardized, commoditized, affordable smart ring product capable of wireless communication across the globe presents a Herculean challenge. However, if introduced according to the above tenets, this could realistically have a marked beneficial impact on global health. As such, it may be attractive to one or more of the emerging cadre of billionaire philanthropists who have demonstrated their capacity to address societal health needs. One could envision the initial development costs being ultimately offset by sponsor nation states, who stand to achieve savings in health care costs while they elevate the standard of care of their citizens [20]. Another potential for revenue funding and also incentivizing subjects to embrace smart ring technology could be the tokenization of personal data for financial gain. Leaving aside the ethical considerations, it is conceivable that trial sponsors or epidemiologists would have interest in the monitoring of data from a large segment of the population for a fixed period of time. This is the basis of compensation for participants in traditional clinical trials, but the scale of the smart ring network globally could offer a quantum leap in opportunities. Such opportunities would need to be carefully structured to adhere to ethical guidelines, but the tactic of sale of personal data is already established in the consumer world, and this could offer a compensatory mechanism that some participants could presumably embrace [21]. Of course, in projecting associated costs globally, it will be important to assess the total economic burden of ownership, which will include device, supply chain, lifecycle updates, and the impact of e-waste. Although future-leaning, the reality of such a device eventually being realized is potentially high. Sensor, wireless, and power technologies are all advancing rapidly, and the key human variable for ring adherence (ie, finger diameter) is relatively narrow (typically 14‐23 mm).

### Technical Hurdles to Be Addressed

In order for the envisioned device to be realized, a number of technical challenges will need to be overcome with regard to device engineering.

#### Network Connectivity

Currently, smart rings transmit data packets via Bluetooth connectivity to smart devices, but the potential for other modalities exist. As described in Figure 2, these include direct-to-cloud, LoRaWAN, and potentially satellite-based systems. A key consideration relates to monetizing these connections, as data continuity is a central component of longitudinal monitoring. While many people enjoy free access to Wi-Fi networks in residences and commercial settings, it may become necessary to provision sponsored services in some situations. For example, patients agreeing to share vital health data to a research organization or sponsor in return for dedicated cellular or Wi-Fi access.

#### Minimum Viable Sensor Units

A key consideration would be the agreement on a minimum set of measures for patient tracking, and the sensors required to enable this. For example, this could be pulse rate, temperature, and actigraphy in a standard battery of assessments. Data could be directly uploaded or stored locally on a miniature hard drive, and then uploaded when network connectivity (low-power wide-area network or bluetooth) was optimal. Given the cost benefits of having a unified model of the device, one solution to facilitate this yet allow additional options to be utilized by other users could be design of a base unit with latent options built in that are only activated on demand. Such product line engineering is often used in the automotive industry, where wiring circuitry is built in on the assembly line independent of final features and options ultimately selected by the customer [22]. In this case, the base and optional features on the ring menu could be selected by state health program needs. Other considerations relate to accuracy and reliability of the sensors themselves, particularly if medical decision-making is intended. Clinical-grade outputs should be a long-term objective for such devices, ideally independent of environmental challenges, such as temperature and humidity fluxes, shock sensitivity, and power surges.

#### Power Supply and Consumption

Advances in the efficiency of rechargeable battery systems continues apace as does miniaturization and improvement in charging cycle intervals [23]. Ideally, the ring charging interval would be >1 month, with the potential either for a cradle-type device or though wireless chargers that are becoming more widely available. Supplementing these systems could be surface-mounted solar-cells that harvest energy for emergency use on demand, for storage on mini-capacitors, or potentially recharging the main battery stack [24]. Other energy-harvesting technologies could be incorporated, including kinetic energy from hand motion, which might be possible through inertial measurement unit sensors [25]. In parallel, refined approaches to circuit design and engineering could serve to reduce power consumption on the device, for example, resting/standby modes that capture and transmit data at defined intervals.

#### Developing Long-term Product Affinity

A key consideration to encourage long-term use in participants will be to arrive at form factors that are functional, esthetically pleasing, and culturally adaptable. Obvious considerations include ring size, material, colors, and the potential for personalization as witnessed in the smartphone market. In theory, and assuming the monetary value was low, the trading-in of rings, for example, at different age points, when BMI changes, or owing to personal preference, could be possible in a mass market, but would require (1) the transaction-style upload of personal health data on demand, and (2) an adequate refurbishment program including the sterilization of the ring. Learning from the smartphone industry would be insightful as a number of repurposing and recycling strategies have been reported [26]. Another means to increase product affinity would be to incorporate dual purpose functionality, for example, adding a security layer that confirmed the person’s identity and thus allowed user access or entry provisions, including entry to buildings [27]. Similarly, the potential for GSM/GPS location tracking functionality could be appealing for family members, school age participants, or care givers.

#### Regulatory Frameworks

**Figure 3.**
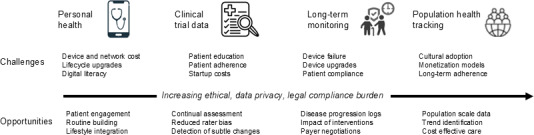
Challenges and opportunities for scaled deployment of digital passive health monitors.

A major consideration for any device used for health tracking will be regulatory compliance. Well-defined pathways have been outlined by health authorities including the United States Food and Drug Administration and The European Medicines Agency on medical devices and the software used to support them. The degree of classification relates to the intended use of data derived, with a naturally high bar for devices used to inform medical decision-making (Figure 3) [28]. Additionally, efforts are underway to utilize the potential of digital devices in support of home care and monitoring, and it can be expected that regulatory policies will evolve as these technologies advance and become more pervasive [29]. Likewise, the sovereignty and privacy of data generated from such devices are subject to governance and regulations, which are often country-specific and require end users to adhere to strict guidelines on capture, storage, use, and accessibility. The deployment of monitoring devices will need to be mindful of current and future regulations, particularly if longitudinal use is desired [30]. Finally, the interoperability frameworks for wireless devices represents another evolving consideration for intended use, with myriad protocols such as FHIR (Fast Healthcare Interoperability Resources), SNOMED CT (Systematized Nomenclature of Medicine – Clinical Terms), and IEEE (The Institute of Electrical and Electronics Engineers) 11073 governing deployment including cross-border applications [31].

### The Pathway to Development

Although a multitude of obstacles need to be addressed, there is evident potential for the deployment of smart ring technologies in long-term health monitoring. In terms of the pathways for development, each of the potential use cases envisioned presents unique challenges and opportunities and the ethical, legal, and data privacy burdens for each are expected to reflect scale and intended use (Figure 3). Nonetheless, given striking advances in health technology, computational and communication networks, and health literacy, it seems inevitable that such frameworks will evolve naturally. In order to propel such opportunities forward, we advocate enhanced dialog between the technology community, health advocacy groups, global health organizations, and philanthropic leaders. We encourage readers of this journal to jump start and advance this dialog as members of the citizen science community, as the impact is potentially significant. Given the evident potential for such a device to play a transformative role in health care reform, we believe this call-to-arms for the device engineering community is timely and warranted and look forward to active dialog to progress this vision.

## Conclusions

Smart ring technology has advanced dramatically over the past decade, and when coupled with new wireless connectivity, offers the potential for use in patient monitoring at individual- through to population-level scales. We posit that by arriving at common standards for design, function, and manufacture, a singular device could be identified for global deployment in health care at a mass scale. To enable such a vision will require active and sustained dialog with myriad stakeholders, seed funding provision from philanthropists, and endorsement from nation member states. Mechanisms for monetization can be envisioned to sustain such an initiative and integrate it into standard health care, and the near- and long-term benefits of this technology could be substantial. The momentum for this may also come through political agendas. A recent vision articulated by the US Health and Human Services Secretary that all Americans wear a digital health device within the next four years may help fuel interest in the approach [32], and the medical community is already providing input on the implementation [33].

## Abbreviations

FHIR: Fast Healthcare Interoperability Resources
GDPR: General Data Protection Regulation
GSM: global system for mobile communication
HIPAA: Health Insurance Portability and Accountability Act
LoRaWAN: low-power area network
SNOMED CT: Systematized Nomenclature of Medicine – Clinical Terms
